# Characterization of Titanium Nanotube Reinforced Cementitious Composites: Mechanical Properties, Microstructure, and Hydration

**DOI:** 10.3390/ma12101617

**Published:** 2019-05-16

**Authors:** Hyeonseok Jee, Jaeyeon Park, Erfan Zalnezhad, Keunhong Jeong, Seung Min Woo, Seungwook Seok, Sungchul Bae

**Affiliations:** 1 Department of Architectural Engineering, Hanyang University, Seoul 04763, Korea; wlgustjr01@gmail.com (H.J.); p9206@hanyang.ac.kr (J.P.); 2 Department of Mechanical Engineering, Biomechacin, Dugas Rd, San Antonio, TX 78251, USA; e.zalnezhad@gmail.com; 3 Department of Chemistry, Nuclear and WMD Protection Research Center, Korea Military Academy, Seoul 01805, Korea; doas1mind@gmail.com; 4 Department of Nuclear Engineering, Texas A&M University, College Station, TX 77843, USA; woosm@tamu.edu; 5 Lyles School of Civil Engineering, Purdue University, West Lafayette, IN 47907, USA; sseok@purdue.edu

**Keywords:** cementitious composite, TNT, nano-reinforcing, mechanical properties, hydration

## Abstract

In recent years, nano-reinforcing technologies for cementitious materials have attracted considerable interest as a viable solution for compensating the poor cracking resistance of these materials. In this study, for the first time, titanium nanotubes (TNTs) were incorporated in cement pastes and their effect on the mechanical properties, microstructure, and early-age hydration kinetics was investigated. Experimental results showed that both compressive (~12%) and flexural strength (~23%) were enhanced with the addition of 0.5 wt.% of TNTs relative to plain cement paste at 28 days of curing. Moreover, it was found that, while TNTs accelerated the hydration kinetics of the pure cement clinker phase (C_3_S) in the early age of the reaction (within 24 h), there was no significant effect from adding TNTs on the hydration of ordinary Portland cement. TNTs appeared to compress the microstructure by filling the cement paste pore of sizes ranging from 10 to 100 nm. Furthermore, it could be clearly observed that the TNTs bridged the microcracks of cement paste. These results suggested that TNTs could be a great potential candidate since nano-reinforcing agents complement the shortcomings of cementitious materials.

## 1. Introduction

Cementitious composites such as mortar and concrete are the most popular and widely-used construction materials all over the world because of their economical and mechanical advantages [1]. However, cementitious composites have major drawbacks such as a brittle nature and poor resistance to crack formation under tensile load even though they possess excellent compressive strength. As viable solutions to overcome these problems, reinforcement techniques in the macro-scale using steel reinforcing bars or various fibers (i.e., steel fiber, synthetic fiber, and carbon glass fiber) [2,3,4,5] have been effectively employed in the cementitious materials. The incorporated fibers shared a part of the tensile force applied to the cementitious matrix, and, after crack occurrence, the fibers continuously resisted the tensile force, which contributed to the improvement of tensile strength and strain capacities [6]. The reinforcing effect of fibers in the cementitious matrix was mostly induced by their high tensile strength [7], elasticity [8], and a geometrically large aspect ratio [6,9,10].

In recent years, nano-reinforcing techniques using 0D nanoparticles, 1D nano-sized fibers or tubes, and 2D nanosheets have opened new possibilities for the reinforcement of cementitious materials [11]. As 0D nanoparticles for cementitious materials, titanium dioxide nanoparticles (nano-TiO_2_) have been a popular material used to improve the physicochemical properties, self-cleaning performance, and photocatalytic effect [12,13,14]. In addition, the nanoparticles can accelerate the hydration of cement phases because of their micro-filling effect and provision of hydration products as nucleation sites [15]. In a number of studies regarding the effects of nano-TiO_2_ on the cementitious composites, Meng et al. [16] found that the early-age compressive strength increased by ~45% with the addition of nano-TiO_2_. In addition, Jayapalan et al. [17] found that nano-TiO_2_ can also accelerate the early-age hydration of tricalcium silicate (C_3_S, Ca_3_SiO_5_) by providing additional spaces for hydration products to grow. Nano-TiO_2_ has also been used to improve the durability of asphalt concrete [18]. In other research fields, nano-TiO_2_ has been widely applied in photocatalysts [15,19,20,21,22], dye-sensitized solar cells [23,24,25], water splitting catalysts for hydrogen production [26], gas sensing [26,27], self-cleaning coatings [28], and superhydrophilicity [29] because it is easy to handle, economical, harmless to the human body, and not affected by temperature. However, as a reinforcing agent for the cementitious materials, nano-TiO_2_ has very little impact on enhancing tensile strength because of its geometrical features [16].

One of the most popular 1D nano-reinforcing material for cementitious materials is carbon nanotubes (CNTs). CNTs generally possess more than 1000 aspect ratios [7,30] and have attracted attention as highly ideal nano-sized reinforcing materials. The reinforcing effect in the interfacial transition zone in the cementitious materials can also be expected with the use of CNTs [31]. CNTs have recently been used to improve the various properties of cementitious materials [32]. However, to date, the main research direction for use of CNTs for reinforcement of cementitious materials has been its improvement effect on the mechanical performance. In the literature with regard to the effect of addition of CNTs on the improvement of the strength of cementitious composites, Campillo et al. [33] found that the addition of single-walled CNTs (SWCNTs) or multi-walled CNTs (MWCNTs) improved the compressive strengths of cementitious materials by 6% and 30%, respectively. Li et al. [34] also observed the strength improvement effect of MWCNTs (~25%) in a 28 days cement mortar composite mixed with 0.5% of surface-modified MWCNTs. Coppola et al. reported that the addition of glass reinforced plastic and CNTs could improve the tensile capacity of cement mortars [35].

Although the CNTs possess great potential as nano-reinforcing materials, some drawbacks have been pointed out in applying them to cementitious materials. The main issue of applying CNTs to cementitious composites is their poor dispersibility because of the inter-tube Van der Waals attraction [36]. A homogeneous dispersion of CNTs is known to be one of the key factors influencing the mechanical performance of CNT-incorporated cementitious composites. If the dispersion of CNTs was sufficient in the composites, the incorporation of a minor addition of MWCNTs of about 0.05 wt.% resulted in an increase of 30% in the flexural toughness of the cementitious composites after 28 days [37]. However, it has been found that obtaining a proper dispersion of CNTs in the cement matrix is challenging because of the large aspect ratio and strong attraction force between the CNTs [38]. The lack of dispersibility of CNTs is known to create many defects in the cementitious composites and reduce the efficiency of the CNTs in the matrix [39]. In addition to the lack of dispersity of CNTs, the hydrophobic characteristic of CNTs may also be a problem leading to a lack of adhesion between the CNTs and the cement matrix [39].

Since Kasuga et al. [40] first reported the synthesis of titanium nanotubes (TNTs) in 1998, they have been widely applied as supports/carriers [41] in the fields of photocatalytic degradation [42], ion exchange, and adsorption [43]. Similar to CNTs, TNTs also possess a large specific surface area [44] and demonstrate high applicability. In addition, Khaled et al. have used TNTs as fillers to reinforce dental resin cements [45]. TNTs have better photocatalytic performance with a larger surface area than TiO_2_ nanoparticles [46]. Therefore, there is also the possibility to enhance the mechanical properties of cementitious materials using less amount of TNTs than TiO_2_ nanoparticles due to its large aspect ratio. 

While TNTs have great potential to serve as nano-reinforcing agents and have been extensively studied for various applications, to the best of our knowledge, there are very few studies of the application of TNTs to cementitious materials. In this study, for the first time, we investigated the quantitative effects of the addition of TNTs on the mechanical properties of cement paste. Properties of hydrothermally synthesized TNTs were characterized using transmission electron microscopy (TEM), X-ray diffraction (XRD), and Brunauer-Emmett-Teller (BET) surface area analysis. Morphological details of fractured surfaces and pore distribution of TNT-incorporated cement paste were observed by using scanning electron microscopy (SEM) and mercury intrusion porosimetry (MIP), respectively. Moreover, the early-age hydration kinetics of TNT-incorporated cementitious composites were also studied by isothermal conduction calorimetry.

## 2. Materials and Methods

### 2.1. Synthesis of TNTs

The TNTs used in the experiments were produced hydrothermally [47]. Titanium tetraisopropoxide (0.01 mol) and acetic acid (0.1 mol) were dissolved in deionized water using a magnetic stirrer (400 rpm) and the mixture was placed in an oven at 70 °C for 72 h. The dried product obtained was crushed into a powder, then transferred into a crucible, and left in the muffle furnace at 450 °C for 2 h. Subsequently, the powder was washed with distilled water five times using a centrifuge at 2000 rpm and then mixed with 55 mL of 10 M NaOH solution and transferred into a Teflon. The autoclave was placed into the oven at 120 °C for 24 h for hydrothermal synthesis. The hydrothermally treated product was washed four times using a centrifuge at 4000 rpm. The rinsed product was then mixed with distilled water in a beaker and HCl (0.1 M) was added to the solution dropwise to attain neutral pH of the mixture (pH = 7). Lastly, the solution was washed five times using a centrifuge and the extracted TNTs from the solution were dried in the oven at 70 °C for 72 h. The crystal structure and specific surface area of the synthesized TNTs were measured by using the XRD and BET method, respectively. 

### 2.2. Specimen Preparation

**Figure 1:**
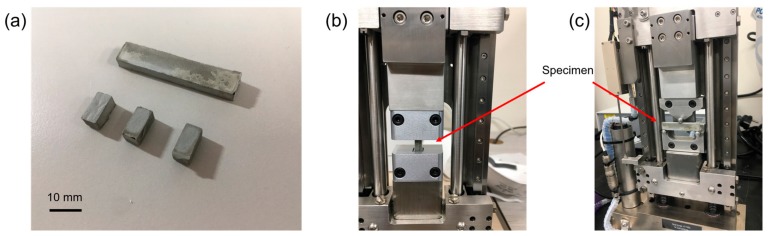
(**a**) Specimens for compressive and bending test, (**b**) compressive test rig, and (**c**) flexural test rig.

Ordinary Portland cement was used to produce the TNT-reinforced cement paste. The chemical composition of the cement was measured by X-ray fluorescence spectroscopy (XRF, ZSX PrimusII, Rigaku, Kashiwa, Japan) and the result is shown in Table 1. The mixing proportions of the specimens are given in Table 2. Neat ordinary Portland cement paste (hereafter referred to OPC) and TNT-reinforced cement paste (hereafter referred to OPC-TNTs) were prepared with water to cement ratios (w/c) of 0.3 and 0.4. For the specimens with w/c = 0.3, a polycarboxylate superplasticizer (ADVA 149, GCP Applied Technologies, Cambridge, MA, USA) was used to gain proper workability. The amount of TNTs added in OPC-TNTs was 0.5 wt.% of the cement component. Prior to cement paste mixing, an aqueous solution of TNTs was prepared. The TNTs were then added to the mixture of distilled water and superplasticizer and subjected to ultrasonic treatment for 5 min using a sonicator (SD-100H, SD Korea Inc., Hwaseong-si, Korea) in order to disperse the TNTs in the solution. Next, this solution was added to cement and mixed in a paste mixer (SPS-1, Select Portfolio Servicing, Inc., Salt Lake City, UT, USA) for 12 min. After 24 h of curing, the pastes were demolded and cured under 20 °C and 60% relative humidity (RH). Figure 1a–c show specimens for compressive and bending test, compressive test rig, and flexural test rig, respectively.

### 2.3. Mechanical Strength Tests

To investigate the mechanical properties of the TNT-incorporated cement paste, compressive and flexural strength tests were conducted using the specimens with w/c of 0.3 and 0.4. Since the amount of synthesized TNTs was limited (~1 g), relatively small specimens were prepared for the mechanical tests. The dimension for compressive strength test were 5 × 5 × 10 mm^3^. For a flexural strength assessment, the three-point bending test was performed on the specimen with dimensions of 4 × 8 × 40 mm^3^. Three specimens were used for each experiment. The compressive strength and flexural strength of the specimens were measured after 1, 3, 7, and 28 days of curing using 5 kN compressive and flexural stages (Microtest, Deben, Suffolk, UK) with the accuracy of 0.01 N. The accuracy of the mechanical test stage using a small paste specimen has been validated in our recent work, which tested the change of relative compressive strength of cement paste with a different amount of nano-TiO_2_ incorporation [48]. 

### 2.4. Mercury Intrusion Porosimetry (MIP) Analysis

Mercury intrusion porosimetry (MIP, Autopore IV 9520, Micromeritics, Norcross, GA, USA) was utilized to investigate the effect of TNTs addition on the total porosity and pore size distribution of the cement paste with w/c = 0.3. The cubic paste samples (5 × 5 × 5 mm^3^) subjected to 28 days of hydration were soaked in isopropanol and diethyl ether to stop further hydration. The treated samples were dried in a 40 °C oven for 48 h prior to the measurements. The maximum pressure was up to 33,000 psia, the density of mercury was 13.5335 g/mL, the contact angle was 130°, and the surface tension of mercury was 0.485 N/m.

### 2.5. BET Analysis

For surface area analysis, the amount of N_2_ gas adsorption was measured at 77 K using a surface characterization analyzer (3Flex, Micromeritics, Norcross, GA, USA). The specific surface area was calculated from the isothermal adsorption curve using the BET equation (Equation (1)).

(1) $$\frac{1}{X\left[\left(P_{0}/P\right)-1\right]}=\frac{1}{X_{m}C}+\frac{C-1}{X_{m}C}\left(\frac{P}{P_{0}}\right)$$

where X is the weight of adsorbed nitrogen at a given relative pressure (P/P_0_), X_m_ is the volume of the single-layer absorption capacity of the gas at a standard temperature and pressure (STP), and C is a constant. STP is defined as 273 K and 1 atm.

### 2.6. Electron Microscopy Analysis

The morphological attributes of the synthesized TNTs were observed by transmission electron microscopy (TEM, JEM 2100F, JEOL Ltd. Tokyo, Japan). To prepare the sample for TEM, an appropriate amount of TNTs was first added to ethanol and ultrasonicated for dispersion. Then, a few drops of the sonicated solution were placed on a carbon film Cu grid and dried in a vacuum desiccator for 48 h. 

The fracture surfaces of OPC-0.3-TNTs (4 × 8 × 40 mm^3^), which were formed after testing the flexural strength at 28 days, were examined by using scanning electron microscopy (SEM, Nova NanoSEM, FEI, Hillsboro, OR, USA). To further clarify the bridge effect of TNTs, samples for SEM were produced by increasing the amount of TNTs (1.5%). SEM was conducted at 15.00 kV and 150,000× magnification. The working distance was 5.8 mm. 

### 2.7. Isothermal Conduction Calorimetry Analysis

Isothermal conduction calorimetry (TAM-air, TA instrument, New Castle, DE, USA) measurements was performed to monitor the effect of TNTs on the early age hydration kinetics of cement and pure C_3_S pastes [49,50]. The w/c of the pastes was set as 0.3 and 0.4 for cement and 0.3 for C_3_S. The total heat of hydration was calculated based on the heat flow data of the isothermal calorimeter. The theoretical model was used to quantitatively explain the effect of the addition of TNTs to C_3_S hydration kinetics (discussed in Section 3) [51,52].

### 2.8. X-Ray Diffraction Analysis 

X-ray diffraction (XRD) analysis was conducted to investigate the effect of TNTs on the hydration products of cement using a D2 Phaser X-ray diffractometer (X-ray source: Cu Kα, λ = 1.5406 Å, range 5° to 70°, step size: 0.01°, step time: 1.5 s, Bruker, Billerica, MA, USA). After the compressive test of OPC-0.3 and OPC-0.3-TNTs at 28 days, fractured paste samples were crushed by a ball mill pulverizer (Pulverisette 23, FRITSCH, Idar-Oberstein, Germany). The fine ground powder that passed through a 400 mesh (38 µm) sieve was then used for XRD. 

## 3. Theoretical Background

Despite the importance of understanding the hydration kinetics of cement, there is no single theory that fully explains its early hydration [52]. The Avrami has been typically used to explain the nucleation growth model of C_3_S. However, challenges of the Avrami model for C_3_S hydration are the assumption that nucleation occurs at randomly distributed locations within the unmodified volume [52]. On the other hand, the kinetics of nucleation and growth under grain nucleation conditions have been mathematically derived by Cahn [51] and referred to as the boundary nucleation and growth (BN) model. Considering a non-transformed volume containing only one plane and assuming that nucleation occurs only at arbitrary positions at this boundary line, a parallel plane at a distance y from the boundary can be envisioned. The cross-sectional area of the nucleated single spherical region at time is then given by Equation (2).

(2) $$\pi \left(G^{2}{\left(t-\tau \right)}^{2}-y^{2}\right)\;\left(if\;t>\tau +\frac{y}{G}\right)$$

where G is the linear growth rate of the transformed phase, t is the time since the start of conversion, and τ is the time when nucleation occurred in the particular region. Equation (3) is the extended region fraction of the intersection region between the curved surface and all regions nucleated at the grain boundaries.

(3) $$Y^{e}=\pi \int_{0}^{t-\frac{y}{G}}I_{B}\left(G^{2}{\left(t-\tau \right)}^{2}-y^{2}\right)d\tau \;(if\;t>\tau +\frac{y}{G})\;Y^{e}=0\left(if\;t<\tau +\frac{y}{G}\right)$$

where I_B_ is the nucleation rate per unit area of the untransformed boundary. Assuming I_B_ is constant, Equation (4) is obtained.

(4) $$Y^{e}=\frac{\pi I_{B}}{3}G^{2}t^{3}\left[1-\frac{3y^{2}}{G^{2}t^{2}}+\frac{2y^{3}}{G^{3}t^{3}}\right]\left(if\;t>\tau +\frac{y}{G}\right)$$

The true area fraction of the intersection area is given by Equation (5), which assumed that nucleation is randomly distributed on the boundary.

(5) $$Y=1-\exp\left(-Y^{e}\right)$$

The volume fraction of a transformed phase originating from a nucleus on a single grain boundary can be determined by integrating the cross-sectional area fraction for all values of the vertical distance between the plane and the interface.

(6) $$X^{b}=2\int_{0}^{\infty }O_{v}^{b}Ydy=2O_{v}^{b}\int_{0}^{Gt}\left[1-\exp\left(-Y^{e}\right)\right]dy$$

where $O_{v}^{b}$ is the boundary area per unit volume and $X^{b}$ is the result of nucleation at a single plane boundary. Next, it is assumed that there are a number of particle boundaries randomly distributed in the original untransformed volume. Then $O_{v}^{b}$ is replaced by the total boundary area per unit volume of $O_{v}^{B}$. The collision from the area originating at the other boundary has not yet been considered. The transformed volume fraction becomes an expanded volume fraction. Equation (7) applies because the boundaries are randomly distributed within the volume.

(7) $$X^{e}=-\ln\left(1-X\right)$$

Combining Equations (6) and (7) gives the true transformed volume fraction for this type of process and the transformation speed can be obtained by differentiating X with respect to time.

(8) $$X=1-\exp\left[-2O_{v}^{B}\int_{0}^{Gt}\left[1-\exp\left(-Y^{e}\right)\right]dy\right]$$

Several minor modifications need to be made to apply Equation (8) to the hydration reaction of C_3_S. The scaling factor A and the time delay parameter t_0_ are applied to the equation. The transformed volume fraction is then expressed as G, $O_{v}^{B}$, and I_B_. However, G, $O_{v}^{B}$, and *I_B_* can be expressed in terms of two independent rate constants, k_G_ and k_B_, in covariance, and the units of both constants are h^–1^, as suggested by Thomas [52].

**Figure 2:**
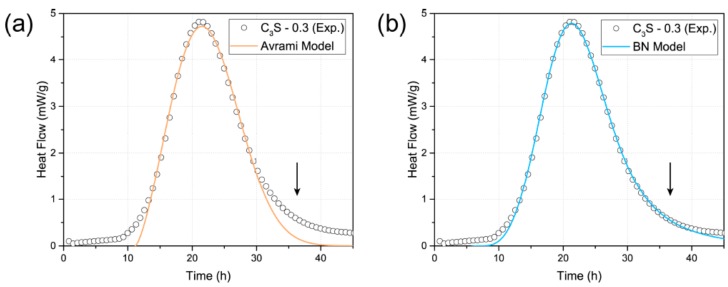
Fitting results of isothermal calorimetry data (○) of C_3_S paste (w/C_3_S = 0.3) using theoretical models of (a) Avrami and (b) BN. Exp.: Experimental data.

(9) kB=IBOvB14G34kG=OvBG

where k_B_ represents the rate at which the nucleated boundary region is transformed. In addition, k_G_ represents the rate at which the non-nucleated particles between the boundaries are transformed. Figure 2 shows the fitting results of C_3_S hydration kinetics using the Avrami model and the BN model. As a result, both models fit well in the acceleration period (~30 h). However, the BN model described the deceleration period (30–40 h) of the hydration curve more accurately than the Avrami model, which was consistent with previous research studies [52,53]. Therefore, we used the BN model to clarify the effects of TNTs on early C_3_S hydration in this study.

## 4. Results and Discussion 

### 4.1. Properties of Synthesized TNTs

**Figure 3:**
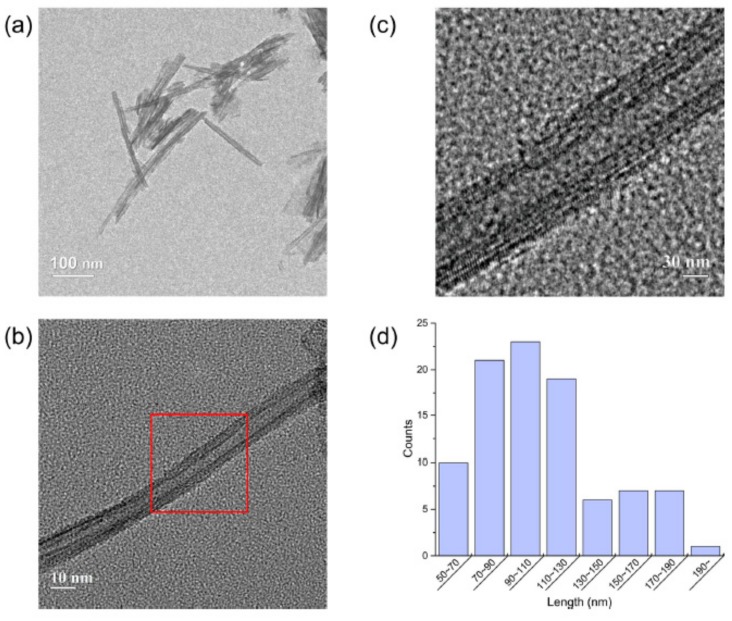
(**a**–**c**) TEM images of hydrothermally synthesized TNTs. The marked area in (**b**) with lower magnification is shown in higher magnification in (**c**). (**d**) Size distribution of TNTs.

The morphology of the hydrothermally synthesized TNTs was examined by TEM, as shown in Figure 3a–c. The size distribution of TNTs in the TEM image was manually calculated (Figure 3d) for about 200 nanotubes. The physical parameters of the observed TNTs are shown in Table 3. The average inner diameter (<I_d_>) and outer diameter (<O_d_>) of the TNTs were ~5 nm and ~11 nm, respectively. In addition, the average length (<l_a_>) of the TNTs was ~100 nm. The calculated aspect ratios of TNTs were approximately 9, in contrast to CNTs with aspect ratios above 1000 [37]. However, due to the relatively smaller size of the TNTs in comparison to CNTs, the effect of filling the pores of cement paste with TNTs may be greater than that of CNTs. Furthermore, a strength enhancement effect is expected because the hydrophilic TNTs exhibit adhesive properties with cement hydration products [29].

**Figure 4:**
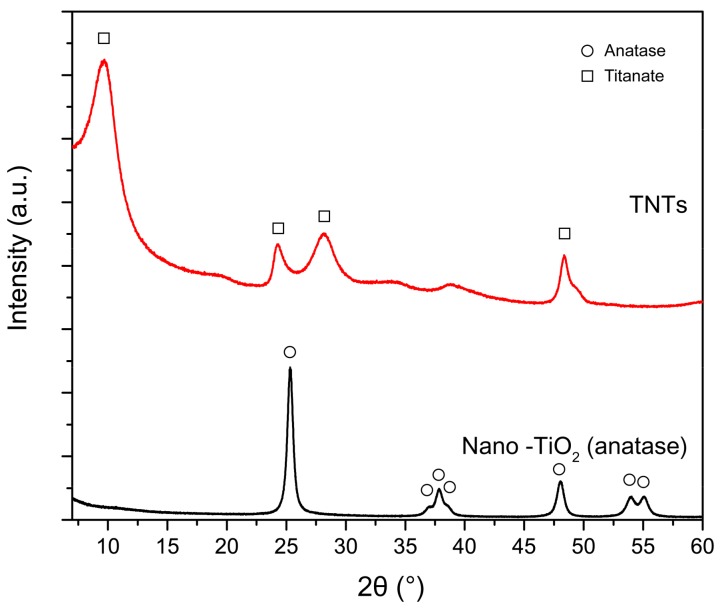
XRD spectra of pure anatase nano-TiO_2_ and TNTs.

Figure 4 displays the XRD patterns of pure anatase nano-TiO_2_ and hydrothermally synthesized TNTs. The XRD pattern of nano-TiO_2_ exhibited diffraction peaks at 25.3°, 36.6°, 36.9°, 37.8°, 48.0°, 53.8°, and 55.0°, corresponding to the anatase phase of TiO_2_ (JCPDS 84-1285) and attributable to the (101), (103), (004), (112), (200), (105), and (211) planes, respectively. The XRD pattern of TNTs showed diffraction peaks at 2θ = 9.6°, 24.6°, 28.0°, and 48.4°, which were attributed to the (200), (110), (310), and (020) crystal planes, respectively, and corresponded to orthorhombic titanate (H_2_Ti_2_O_5_) (JSCPDS 47-0124). This XRD pattern was consistent with that of the TNTs synthesized previously via a rapid hydrothermal method by An’amt et al. [44]. In particular, the intensity of the peak at 2θ = 9.6° was clearly high. The corresponding d-spacing of this peak was 0.93 nm, which corresponded to the multilayered-wall interlayer distance of the TNTs [44].

**Figure 5:**
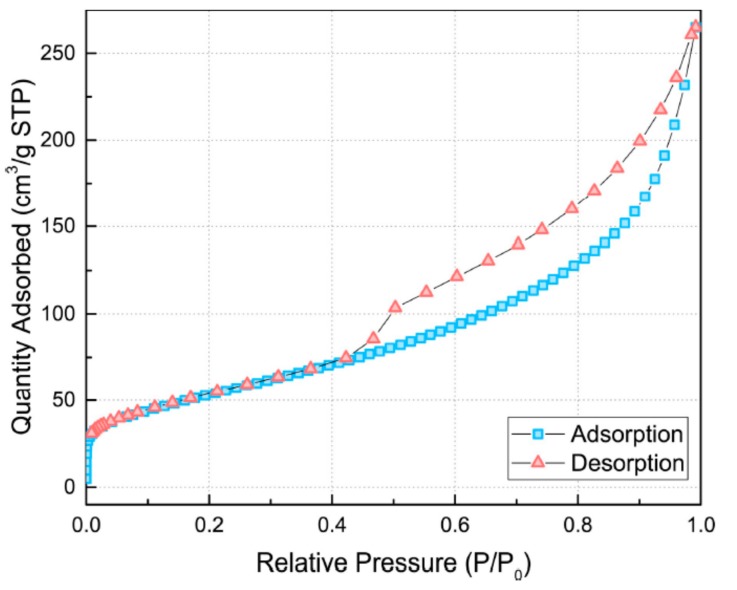
N_2_ adsorption isotherm for the hydrothermally synthesized TNTs.

The nitrogen (N_2_) adsorption isotherm of the synthesized TNTs is exhibited in Figure 5. The isotherm is IUPAC (International Union of Pure and Applied Chemistry) type IV pattern with a hysteresis loop and has a sharp inflection in the N_2_ adsorbed volume at P/P_0_ of ~0.45, which indicates that the TNTs are present in tubular structures. The BET surface area of the TNTs was calculated to be ~200 m^2^/g, which is about four times than that of untreated nano-TiO_2_ [54]. In the case of CNTs, the specific surface area ranges from 90 to more than 500 m^2^/g [30,55,56,57,58].

### 4.2. Effect of TNTs on the Hydration of Cement Paste and Tricalcium Silicate (C_3_S) 

**Figure 6:**
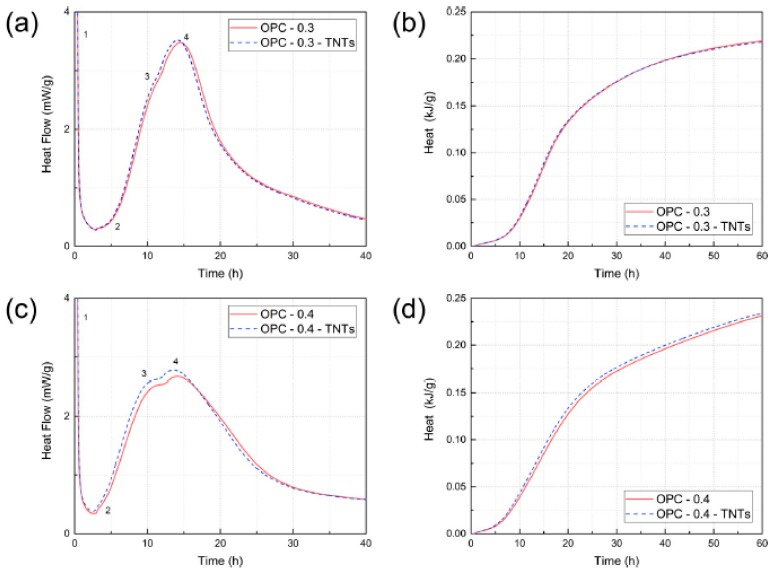
Rate of hydration measured by isothermal conduction. (**a**) Heat flow of OPC-0.3 with and without TNTs. (**b**) Total heat of hydration values calculated by integration of data in (**a**). (**c**) Heat flow of OPC-0.4 with and without TNTs. (**d**) Total heat of hydration values calculated by integration of data in (**c**).

The results of isothermal conduction calorimetry to determine the effect of TNTs on hydration of cement and C_3_S are shown in Figure 6. The numbers in Figure 6a,c indicate the peaks as: (1) initial C_3_A reaction, (2) C_3_S surface reactions at the end of the induction period, (3) the main C_3_S hydration peak, and (4) ettringite ((CaO)_6_(Al_2_O_3_)(SO_3_)_3_·32H_2_O) formation, respectively [59]. 

Figure 6a shows the isothermal conduction calorimetry results for w/c = 0.3 samples. The results show that C_3_S and ettringite formation of OPC-0.3-TNTs was slightly accelerated as compared to OPC-0.3. However, in Figure 6b, which depicts the heat of hydration values calculated by the integration of heat flow shown in Figure 6a, the overall heat evolution of hydration was not different. Similar results were obtained with the w/c = 0.4 sample without the use of a superplasticizer. Figure 6c,d show the heat flow and the overall heat evolution of w/c = 0.4 samples, respectively. Similar to the previous results, the initial surface activity of the OPC-0.4-TNTs sample was slightly faster, but the total heat of hydration was not significantly different.

**Figure 7:**
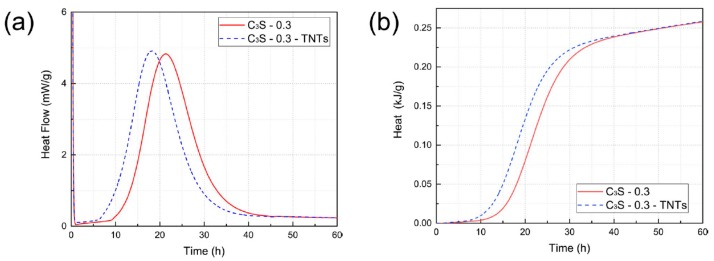
(**a**) Heat flow of C_3_S with and without TNTs measured by isothermal conduction calorimetry. (**b**) Heat of hydration calculated by integration of data in (**a**).

Pure C_3_S has been widely used as a hydration kinetics model system of Portland cement [52]. The results of isothermal conduction calorimetry of C_3_S with and without TNTs are shown in Figure 7. In the case of C_3_S samples, there were clearly different results compared to the OPC samples. The presence of TNTs accelerated the C_3_S hydration peak by ~5 h. However, the total heat of hydration shown in Figure 7b was not significantly different compared to the OPC results.

The degree of hydration (α) was calculated after assuming the hydration enthalpy of C_3_S to be 121 kJ/mol [60]. The degree of hydration after 50 hours of hydration was calculated to be 55.6% for pure C_3_S and 55.7% for TNT-incorporated C_3_S mixture. This implied that TNTs accelerated the hydration of C_3_S in the early stages but did not significantly affect its degree of hydration. Previous studies have shown that cement hydration can be accelerated when fine inert materials are added to the cement [17,61,62]. Jayapalan et al. [17] showed that cement hydration was accelerated when TiO_2_ nanoparticles were added to Portland cement, and the effect was reportedly quite large when the size of TiO_2_ was small. Zhang et al. [62] also reported that the addition of 5% TiO_2_ could accelerate the hydration of the cement. This implied that the added materials acted as seeding materials in the hydration reaction of cement. According to Thomas et al., when a relatively small amount of C-S-H was added to Portland cement or pure C_3_S, the reaction was accelerated by the seeding effect of C-S-H [17,60,61].

**Figure 8:**
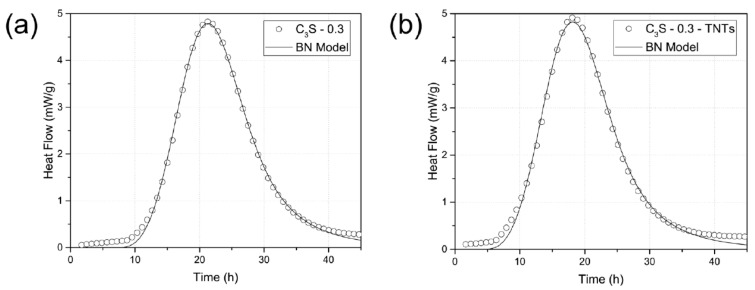
Standard boundary nucleation and growth fitting (solid line) to the C_3_S hydration kinetic data (○). (**a**) C_3_S-0.3, (**b**) C_3_S-0.3-TNTs.

The hydration kinetics were modeled using the BN model to further study the experimental results for pure C_3_S. Figure 8 shows the fitting results of the initial hydration behavior of C_3_S using the BN model, and the related parameters are shown in Table 4. The fitting data is obtained by the least square method. From the modeled parameters, when 0.5 wt.% TNTs were added to C_3_S, k_B_ increased by 0.00105 from 0.06314 to 0.06419, which is an increase of about 1.7%. On the other hand, k_G_ increased by ~1.9% from 0.06262 to 0.06381. k_B_ describes the transformation rate at the grain boundary (particle surface), while k_G_ describes the rate of transformation at the non-nucleated particles (intergranular void space). The ratio of k_B_ to k_G_ among the modeling parameter indicates the type of kinetic behavior. When k_B_/k_G_ is large, then the transformation occurred prematurely in the overall process [52]. A slight reduction in k_B_/k_G_ could be explained by the delay of the start of diffusion-controlled hydration kinetics and the increase of the amount of early nucleation and growth hydration, due to the presence of TNTs providing additional nucleation sites away from the C_3_S particles [60]. However, the very small variance of k_B_/k_G_ confirmed that the TNTs did not significantly affect the overall hydration of the cement paste. Meanwhile, when compared to the control specimen, the parameter t_0_ decreased by 2.93 h when TNTs were added. The decrease in t_0_ was found to shift the fit of the BN model to the left, which indicates that the TNTs had a promoting effect only at the very early reaction stage of the cement paste [53].

Previous studies have also shown that the addition of nanoparticles such as TiO_2_ nanoparticles or CNTs accelerates the hydration of cement and C_3_S [53,59,61]. Lee et al. showed that the addition of TiO_2_ nanoparticles with a large surface area provided nucleation sites that accelerated the hydration reaction [53,59,61]. However, k_B_/k_G_ tended to decrease by the addition of 5 wt.% of TiO_2_ [53]. This experiment showed that the addition of a relatively small amount of TNTs (0.5 wt.%) had a significant effect on the hydration kinetics compared with the existing studies. Moreover, it was found that the BN model properly represented the hydration kinetics of the C_3_S-0.3 and C_3_S-0.3-TNTs systems.

**Figure 9:**
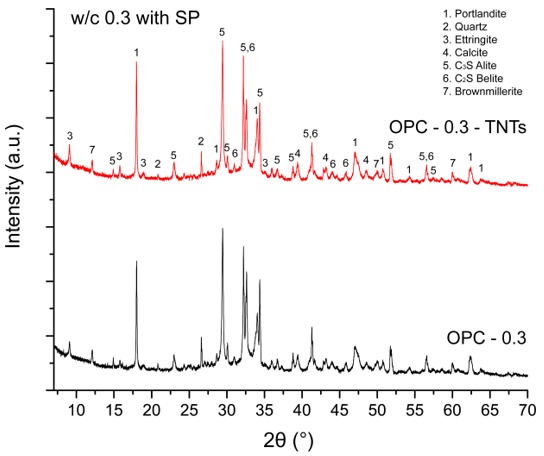
XRD patterns of OPC-0.3 and OPC-0.3-TNTs at 28 days of curing.

Based on the isothermal calorimetry and theoretical modeling, we found that the effect of TNTs on the total hydration degree of cement paste at 60 hours was minor. To investigate the effect of TNTs on the hydration product, XRD analysis was conducted for sample curing for 28 days (Figure 9). The results showed that the main peak of Ca(OH)_2_ (2θ = 18.1°) had slightly increased with the addition of TNT but there was little effect on the total hydration. This observation was consistent with the calorimetry results that a small amount of TNTs did not significantly affect the hydration of cement paste after the deceleration period.

### 4.3. Mechanical Properties and Microstructure of TNTs-Cement Composites

**Figure 10:**
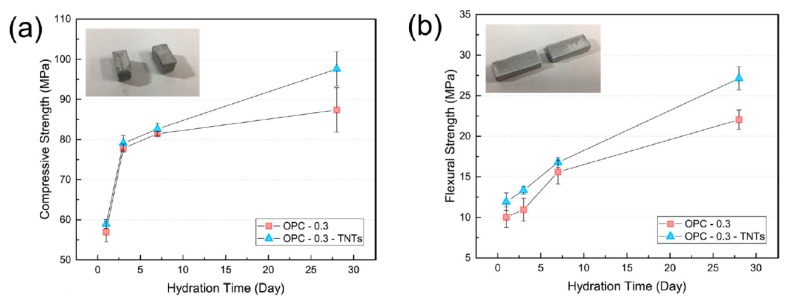
(**a**) Compressive and (**b**) flexural strength of ordinary Portland cement paste with TNTs (w/c = 0.3).

Compressive and flexural strengths were measured to determine the effect of reinforcement by TNTs on the mechanical properties of cement paste. In general, nanoparticles are able to fill the pores and provide a compact microstructure [63]. The variations of compressive strength and flexural strength of OPC-0.3 and OPC-0.3-TNTs at 1, 3, 7, and 28 days are displayed in Figure 10. The compressive strength of TNT-reinforced samples was higher than that of neat ordinary Portland cement paste (OPC-0.3) at all curing days. Especially, the compressive strength of OPC-0.3-TNTs was remarkably increased (11.7%) after 28 days. Figure 10b shows the three-point flexural strength of OPC-0.3 and OPC-0.3-TNTs with a superplasticizer. Flexural strength was also enhanced because of TNT-reinforcement by 19.0%, 21.6%, 7.3%, and 23.15% after 1, 3, 7, and 28 days, respectively. As with the compressive strength, the flexural strength was also significantly improved after 28 days. 

**Figure 11:**
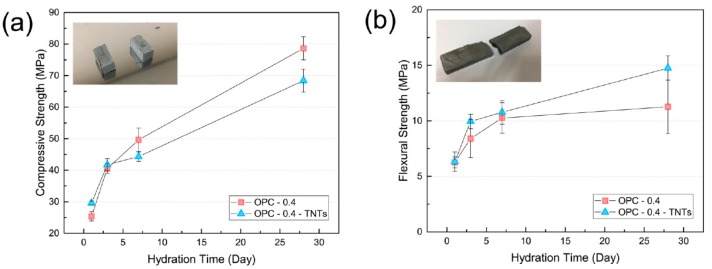
(**a**) Compressive and (**b**) flexural strength of ordinary Portland cement paste with TNTs (w/c = 0.4).

The compressive and flexural strengths of TNT-reinforced cement paste of w/c = 0.4 without using superplasticizer are shown in Figure 11. Unlike the results of the cement paste having w/c = 0.3 with the superplasticizer (described above), the compressive strength of OPC-0.4-TNTs was comparatively lower than that of OPC-0.4 after 7 and 28 days and was reversed at w/c = 0.4. As a result, the compressive strength of OPC-0.4-TNTs was increased by 16.6% after one day and 3.0% after three days compared to OPC but decreased by 10.7% after seven days and 13.0% after 28 days. In contrast to the compressive strength results, the flexural strength had increased for all curing days, i.e., 1.2%, 18.2%, 5.0%, and 31.4% after one, three, seven, and 28 days, respectively. 

The specimen at 28 days of curing possessed larger error values of compressive and tensile strength compared to those measured at early ages. The implication of the large variation of compressive strength at 28 days of curing could not be clarified in this study. However, we assumed that the large error values were due to the effect of curing condition (60 % RH) because the degree of cement hydration was limited and relatively large pores (>37 nm) were formed in cement paste when it was cured at low RH (≤81%) [64].

In previous studies, it has been shown that CNTs have positive effects on the mechanical properties of cementitious materials. Konsta-Gdoutos et al. have reported that cement paste containing CNTs increased the rate of production of calcium silicate hydrate (C-S-H) gels with high stiffness because of CNTs incorporation [37]. In addition, CNTs having a large specific surface area per unit volume promoted the formation of hydrates by performing the role of a generating nucleus in which the cement hydrate was generated [59]. Additionally, nano-sized, fine CNTs, which have a larger size than the TNTs described in this study, could fill the micropores between C-S-H gels to reduce porosity [63]. As with CNTs, it was evident that TNTs also possessed the ability to enhance the mechanical properties of cement paste, especially at long-term age. However, it is speculated that the decrease in compressive strengths of w/c = 0.4 samples after 7 and 28 days was caused by the absence of the superplasticizer. The superplasticizer could help the dispersion of the TNTs in the cement paste, which results in the enhancement of strength of the cement matrix. Previous studies also have shown that the dispersion of CNTs had a major influence on the strength of the cement matrix and the presence of surfactant could help the dispersion of the CNTs [37,65,66].

**Figure 12:**
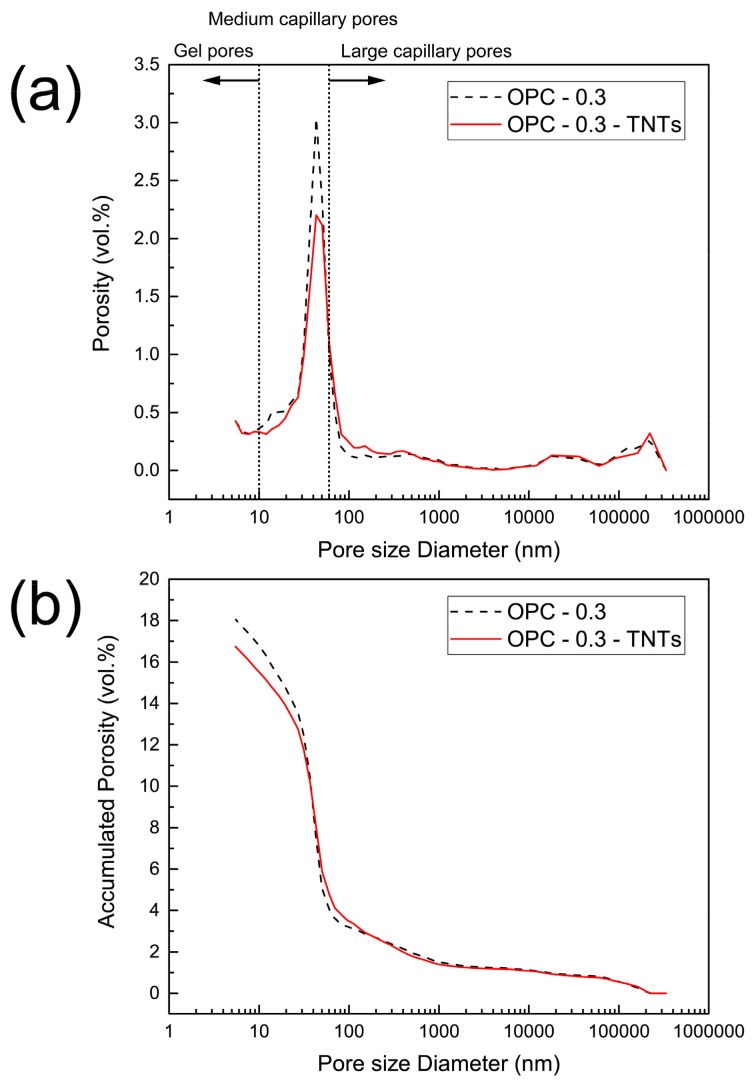
MIP results of ordinary Portland cement paste with TNTs, (**a**) pore size distribution, and (**b**) total porosity.

Pore analysis of cement paste with and without TNTs was performed to further investigate the influence of porosity and pore size distribution on the strength of OPC-0.3 and OPC-0.3-TNTs after 28 days (Figure 12). Previous studies have classified the pore size of hydrated cement paste into three types: 50–10,000 nm, 10–50 nm, and <10 nm represent large capillary pores, medium capillary pores, and gel pores, respectively [67]. It was found that TNT-incorporation decreased the medium capillary pores in the cement paste (Figure 12a). The average pore size did not show a significant difference. The average pore radius of OPC-0.3 and OPC-0.3-TNTs were 30.4 nm and 31.4 nm, respectively. However, it is shown that accumulated pore volumes of OPC-0.3-TNTs was decreased (Figure 12b). Compared to OPC-0.3, a 7.11% decrease occurred in the paste containing TNTs. Similar results have been observed previously with MWCNTs cement composites by Nochaiya et al. [63]. The results showed that the total porosity of the mixture of CNTs and cement decreased as the content of CNTs increased, which supported the increase in compressive strength of the cement composites because of the filling of CNTs between the hydration products of cement [63]. Therefore, the enhancement of compressive strength of the OPC-0.3-TNTs could be explained by the relatively small size of TNTs filling the pores of cement hydration products.

**Figure 13:**
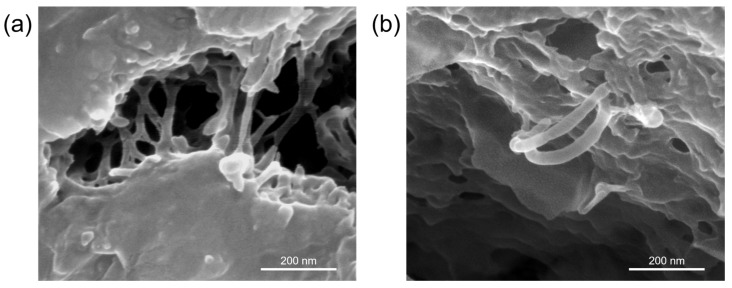
(**a**,**b**) SEM images of the crack bridging effect of TNTs in ordinary Portland cement paste at different locations of the fractured specimen.

Figure 13a,b show the SEM image of the cracked surface of OPC-0.3-TNTs. Considering the small size of TNTs, a larger amount of TNTs (1.5%) was added into the paste as compared to that added in the specimen for testing the flexural strength in order to increase the possibility to find TNTs. In the SEM image, it was clearly observed that the TNTs bridged the nano-sized cracks that appeared between the hydration products in the cement paste. This showed the crack-bridging effect of TNTs even though the aspect ratio was relatively small compared to that of CNTs. According to Nadiv et al., the straight shape of the nanotubes was found to reinforce cementitious composites more efficiently than the wave shape due to the longer effective length [68]. It is assumed that relatively small but straight shape TNTs effectively reinforce the nano-sized cracks in the cement matrix. Moreover, it is worth noting that the average thickness of TNTs in the cement paste increased to 22 nm, as compared to those before mixing with cement (<O_d_> ≈ 11 nm). The increased thickness was induced by cement hydrates such as C-S-H tightly enclosing the TNTs. The surface of the hydrophilic TNTs [69] provided better sites for the cement hydrates, which allowed for enhanced bonding strength between the TNTs and cement paste. Thus, it was demonstrated that the bridging effect provided by TNTs improved the flexural strength of the cement paste prepared in this study.

## 5. Conclusions

In this study, the effect of incorporating TNTs in cement pastes on their mechanical properties, microstructure, and hydration kinetics were investigated for the first time. The obtained results can be summarized as follows.

(1) Analysis of the mechanical properties of cement pastes with added TNTs confirmed the reinforcing effect of the TNTs. The TNTs increased the strength of the cement pastes at most ages and particularly the later age strength. Thus, addition of TNTs was considered to be more effective for increasing flexural strength than compressive strength. The strength enhancement was a result of the nano-sized fine TNTs decreasing the porosity of cement paste in the pore size range of 10 to 100 nm. The SEM measurements of the fractured surfaces of cement paste clearly showed the bridge effect of TNTs between the microcracks of cement paste.
(2) In the case of ordinary Portland cement paste, the initial hydration properties of the paste did not show any significant change with the addition of TNTs regardless of the w/c ratio. In contrast, in the case of C_3_S, it was confirmed that the addition of TNTs accelerated the hydration by several hours. There was no significant difference in the total hydration rate between the control and TNT-incorporated samples.

Based on these results, it was concluded that TNTs have the potential as nano-reinforcing materials for cementitious materials. In the near future, we aim to study the effect of the amount of incorporated TNTs, their size, and aspect ratios on the properties of cementitious materials. The quantitative effect of the hydrophilic surface of TNTs also needs to be studied.

## Figures and Tables

**Table 1:** Chemical composition of ordinary Portland cement.

SiO2	Al2O3	Fe2O3	CaO	MgO	K2O	SO3	TiO2	LOI *	Total
18.43	2.83	2.17	68.17	2.37	1.11	3.03	0.15	1.72	100

* LOI: Loss of ignition.

**Table 2:** Mixing proportions of TNTs/OPC.

Sample	w/c *	TNTs	Superplasticizer
OPC-0.3	0.3	0	0.40 wt.%
OPC-TNTs-0.3	0.3	0.50 wt.%	0.40 wt.%
OPC-0.4	0.4	0	0
OPC-TNTs-0.4	0.4	0.50 wt.%	0

* w/c: Water to cement ratio.

**Table 3:** Physical parameters of TNTs.

Products	Outer Diameter (nm)	Inner Diameter (nm)	Length (nm)	Surface Area (m^2^/g)
**TNTs**	11 ± 1.9	5 ± 0.8	100 ± 36	~200

**Table 4:** Fitting parameters obtained by applying the boundary nucleation and growth models to C_3_S-0.3 and C_3_S-0.3-TNTs hydration data.

Specimen	A (kJ/mol)	t_0_ (h)	k_B_ (h^−1^)	k_G_ (h^−1^)	k_B_/k_G_
**C_3_S- 0.3**	67.47	7.12	0.06314	0.06262	1.0083
**C_3_S-0.3-TNTs**	66.76	4.19	0.06419	0.06381	1.006
